# Data envelopment analysis efficiency in the public sector using provider and customer opinion: An application to the Spanish health system

**DOI:** 10.1007/s10729-021-09589-7

**Published:** 2022-02-01

**Authors:** Jesús A. Tapia, Bonifacio Salvador

**Affiliations:** Department of Statistic and Operative Research, University of Valladolid, Campus Miguel, Delibes, Paseo Belén nº7,47011 Valladolid, Spain

**Keywords:** Data envelopment analysis, Sampling survey research, Public sector, Sample size, Bootstrap, Efficiency confidence interval

## Abstract

Measuring the relative efficiency of a finite fixed set of service-producing units (hospitals, state services, libraries, banks,...) is an important purpose of Data Envelopment Analysis (DEA). We illustrate an innovative way to measure this efficiency using stochastic indexes of the quality from these services. The indexes obtained from the opinion-satisfaction of the customers are estimators, from the statistical view point, of the quality of the service received (outputs); while, the quality of the offered service is estimated with opinion-satisfaction indexes of service providers (inputs). The estimation of these indicators is only possible by asking a customer and provider sample, in each service, through surveys. The technical efficiency score, obtained using the classic DEA models and estimated quality indicators, is an estimator of the unknown population efficiency that would be obtained if in each one of the services, interviews from all their customers and all their providers were available. With the object of achieving the best precision in the estimate, we propose results to determine the sample size of customers and providers needed so that with their answers can achieve a fixed accuracy in the estimation of the population efficiency of these service-producing units through the use of a novel one bootstrap confidence interval. Using this bootstrap methodology and quality opinion indexes obtained from two surveys, one of doctors and another of patients, we analyze the efficiency in the health care system of Spain.

## Introduction

Data Envelopment Analysis (DEA) has become a widely used technique to compare the efficiency of service-producing units because it easily handles the multiple outputs characteristic of public sector production, is non-parametric and does not require input price data [34]. The popular application is with deterministic information and classic DEA models [3, 10], for instance, in health care [1, 37] or [25]), universities and research institutes [26], government [17], public libraries [22, 23], schools [35], public transport services [15] or banks [28].

In recent years, some approaches consider the opinion of the customer to be crucial for measuring DEA efficiency in public services ([5, 16]). Consumer satisfaction-opinion surveys are a common tool for building opinion indexes, which measure the quality of the service, and to be used as output variables in DEA ([20, 27, 31, 32, 35, 40, 41] and [42]).

Besides customer opinion-satisfaction (output), provider opinion-satisfaction (input) is also a fundamental protagonist in public service-producing units (SPUs), our decision-making units. The provider’s positive opinion-satisfaction concerning the service offered may result in a better customer opinion concerning the service received. For instance, a city’s public bus drivers with a good opinion of their salary, timetable, partners, driven vehicles, etc., may influence a better satisfaction-opinion of the travellers using the service. One way to measure the service quality offered is with satisfaction-opinion indexes obtained through survey samples carried out with the providers. In Tapia et al. [40–42] the protagonism of the opinion of the providers is not considered, that is, the inputs are deterministic and only the outputs are estimated using a customer sample. Introducing the opinion-satisfaction of the providers as estimated indexes increases the field of application of this work, where opinion-satisfaction indexes, estimated from a sample of providers (inputs) and a sample of customers (outputs), are used as information to measure the DEA efficiency of the public services. To do so, it is necessary to determine the sampling design, the sample size and the estimators of the satisfaction-opinion indexes of both the providers and the customers in each SPU.

In practice, this stochastic input and output information is available in many services, as it is increasingly common to conduct opinion-satisfaction surveys for both providers and customers. For instance, the sample data used in the Spanish health system application of Section 5.

The efficiency obtained with DEA models, using the opinion indexes estimated with the survey answer of samples of providers and consumers as data, will be an estimation of the population DEA efficiency. This population efficiency is an unknown non-evaluable parameter, since it would be necessary to use the indexes obtained with the opinion of all the customers and all the providers of all the services as data and this is a census of the entire population [40]. This statistical analysis of the DEA efficiency gives rise to the problem of determining the sample size of customers and providers needed to guarantee an a priori fixed accuracy in the estimation, with confidence interval, of the DEA efficiency of each public service, which is the object of our investigation. Liu et al. [30] considered the statistical analysis and sampling process as an important direction for handling the DEA. Ceyhan and Benneyan [7] investigated the impact of the sample size on the measures of efficiency when the DEA problem was carried out on values that include such estimated proportions as defect, satisfaction, mortality, or adverse event rates estimated from samples. Nevertheless, they do not propose a solution to the necessary sample size in order to control the error in the measures of efficiency. The problem of calculating the sample size of customers necessary when the objective is to estimate, with a fixed precision in the estimate error, the population efficiency in a finite set of public services using stochastic data output (customer opinion-satisfaction indexes) and known (non-stochastic) data input was resolved in Tapia et al. [40, 42].

In this paper, we propose a solution to determine the customer and provider sample sizes needed to estimate the DEA efficiency in public services with bootstrap confidence intervals and a fixed accuracy. These intervals capture the random variations introduced in the DEA analysis by using outputs and inputs estimated with a sample. So far, Simar and Wilson’s [38, 39] methodology has been the most common for measuring efficiency with bootstrap confidence intervals in such public services as health care ([11]), universities and research institutes [4], government [6], public libraries [29], schools [19], tourism [2], banks [28] or public transport services [21]. The problem posed by Simar and Wilson is different from ours because the stochastic character of the inputs and outputs is different. In Simar and Wilson, the stochastic character of the input and output information comes from considering the set of available SPUs, *S*_*n*_, as a sample from an infinite population and the sample observations in *S*_*n*_ are realizations of identically, independently distributed (iid) random variables with a probability density function with support over $P=\left \lbrace (x,y) | x \text { can produce } y\right \rbrace $, [14]. In this work, the set of available SPUs are the only units whose efficiency we wish to evaluate. Therefore, we do not consider them a sample as in the classical DEA models, [9]. The stochastic character of our output and input information comes from the fact that these data are opinion-satisfaction indexes that it is necessary to estimate by taking independent random samples in each SPU, and the probability, in the statistical model, depends on the sample design used in each SPU.

In Shwartz [37], a random sample of patients arriving for health care services were bootstrap resampled to obtain data input and interval estimates of the DEA efficiency. These interval estimates are conservative (large) and the problem of the patient sample size necessary to obtain the desired accuracy in the estimation of the DEA efficiency is not resolved.

In other approaches, where samples in public services are used to estimate the data (Charles2014), the efficiency is estimated with stochastic DEA models. These models, which use LP problems subject to constraints defined in terms of probability, are also called chance-constrained problems. The deterministic characterization of “efficient” is then changed by the probabilistic characterization “probably efficient”. A vast number of papers show a wide range of uses of chance-constrained programming (CCP), including [8, 9, 12, 33] or [43]. One of the main advantages of the technique presented in this study is its simplicity, as we use the original DEA models with constant (CCR) and variable returns-to-scale (BCC), that is, linear programming (LP) problems subject to deterministic constraints [3, 10].

In this paper, we therefore examine the implications of input and output data estimated with a provider and a customer sample, respectively, for the performance analysis of public services using DEA analysis techniques. In Section 2, we examine the nature of the problem. Section 3 presents a theoretical method to determine the customer and provider sample size needed to estimate the population DEA efficiency with bootstrap confidence interval, which is examined in Section 4. Section 5 includes the empirical application in the Spanish health system we have undertaken. Section 6 contains our conclusions. All the software is our own elaboration using MATLAB and it is included in Appendix A.

## Nature of the problem

We consider a finite fixed set of *L* SPUs, service-producing units, one provider interview to estimate *m* inputs and one customer interview to estimate *s* outputs. For example, in each hospital of a homogeneous set of *L*, it is possible to estimate the general satisfaction of the personnel, or their annual time of formation, or other personal opinion indexes (input data) by interviewing a sample of the personnel. It is also possible to estimate the satisfaction of the patients attention they have received, the human and material resources of the hospital, etc., by interviewing to a patient sample (output data). The main problem approached in this paper is to estimate the unknown parameter population DEA efficiency. The population efficiency, or census efficiency, is unknown because, in order to know it, it would be necessary to interview all the providers and all the customers (census) of each one of the *L* public services and, with this population data, to obtain the opinion indexes and use them as input/output data in the classic LP model CCR or BCC of Table 1; in real applications, these censuses are completely non-viable.

**Table 1:**
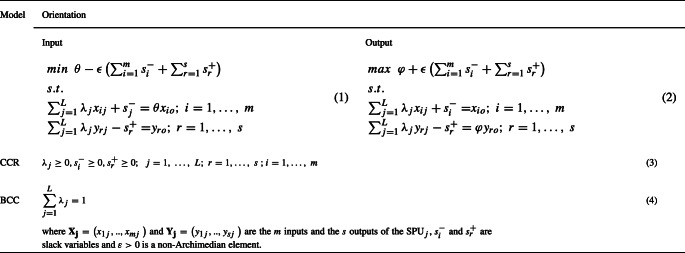
DEA models with variable (BCC) and constant returns-to-scale (CCR)

The error made in estimating the input/output data with samples is transferred to the DEA efficiency estimation. In this study, we propose a methodology that guarantees a reasonable quality of the DEA efficiency estimation.

Formally, in the jth service-producer unit (SPU_*j*_ for short), we consider the finite provider population ${\mathbf {U_{j}}}= \left \{{{\mathbf U}}_{{\mathbf 1}{\mathbf j}},{\dots } ,{{\mathbf U}}_{{{\mathbf N}}_{{\mathbf {x_{j}}}{\mathbf j}}} \right \} $ of size $N_{x_{j}}$ and the customer population $ {\mathbf {W_{j}}}=\left \{{{\mathbf W}}_{{\mathbf 1}{\mathbf j}},{\dots } ,{\mathbf W}_{\mathbf {{N_{y_{j}}} j}} \right \} $ of size $N_{y_{j}}$. Each provider is a quantitative vector ${{\mathbf U}}_{{\mathbf {kj}}} =\left (U_{k1j},{\dots } ,\ U_{kmj}\ \right )$, where *U*_*kij*_ is the answer of the kth provider of the jth SPU to the ith opinion provider item, i.e., each customer ${{\mathbf W}}_{{\mathbf { hj}}}=\left (W_{h1j},{\dots } ,\ W_{hsj}\ \right )$ is a quantitative vector where *W*_*hrj*_ is the answer of the hth customer of the jth SPU to the rth customer opinion item. The population input and output data are opinion indexes obtained as a function of the provider and customer population answers, in general:

5 $$ \begin{array}{@{}rcl@{}} \mathbf{X_{j}} &= & \left( f(U_{11j}, \cdots,U_{{N_{x_{j}}}1j}), \dots, f(U_{1mj}, \cdots,U_{{N_{x_{j}}}mj}) \right) ; \ j = 1,\dots, L \end{array} $$

6 $$ \begin{array}{@{}rcl@{}} \mathbf{Y_{j}} &= & \left( g(W_{11j}, \cdots,W_{{N_{y_{j}}}1j}), \dots, g(W_{1sj}, \cdots,W_{{N_{y_{j}}}sj}) \right) ; \ j = 1,\dots, L \end{array} $$

These functions *f* and *g* can be of any type, with or without weights, whenever they admit an interpretation of *m* population opinion-satisfaction indexes as input and *s* population opinion-satisfaction indexes as output.

Using the data ${\left \lbrace \left (\mathbf {X_{j}}, \ {{{\mathbf Y}}}_{\mathbf j}\right )\right \rbrace }_{j=1,{\dots } ,L}$ in the Table 1 models, we obtain the population DEA efficiency scores $ {\left \lbrace \varphi _{j} \right \rbrace }_{j=1, ..., L} $. If it were possible to carry out a census and to know these population indexes, the information would be fixed or deterministic and the character of the problem would not be stochastic.

A lack of knowledge concerning the population information input and output makes the taking of samples to estimate ${\left \lbrace \varphi _{j} \right \rbrace }_{j=1, ..., L} $ necessary. In the SPU_*j*_, let $ \left \{{{\mathbf U}}_{{\mathbf 1}{\mathbf j}},{\dots } ,{{\mathbf U}}_{\mathbf {n_{x_{j}} j}} \right \}\subset {\mathbf {U_{j}}} $ and $ \left \{{{\mathbf W}}_{{\mathbf 1}{\mathbf j}},{\dots } ,{{\mathbf W}}_{\mathbf {n_{y_{j}} j}}\right \} \subset {\mathbf {W_{j}}}$ be random samples of size $N_{x_{j}}$ and $N_{y_{j}}$ of the provider and customer populations, respectively. To obtain the estimators of the input indexes Eq. 5 and the output indexes Eq. 6, the same functions *f* and *g* are used with the random samples:

7 $$ \begin{array}{@{}rcl@{}} \mathbf{{\widehat{X}}_{j}} &= & \left( f(U_{11j}, \cdots,U_{{n_{x_{j}}}1j}), \dots, f(U_{1mj}, \cdots,U_{{n_{x_{j}}}mj}) \right) ; \ j=1,\dots, L \end{array} $$

8 $$ \begin{array}{@{}rcl@{}} \mathbf{{\widehat{Y}}_{j}}&= & \left( g(W_{11j}, \cdots,W_{{n_{y_{j}}}1j}), \dots, g(W_{1sj}, \cdots,W_{{n_{y_{j}}}sj}) \right) ; \ j=1,\dots, L \end{array} $$

Having observed the provider and customer sample answers $\left \lbrace {\mathbf {u_{kj}}= \left (u_{k1j}, \dots , \ u_{kmj}\right ) }\right \rbrace _{ {k=1,{\dots } ,{n_{x_{j}}}} }$ and $\left \lbrace {\mathbf {w_{hj}}=\left (w_{h1j}, \dots , \ w_{hsj}\right ) }\right \rbrace _{ {h=1,{\dots } ,{n_{y_{j}}}} }$, respectively, Eqs. 9 and 10 are the estimates of the input and output indexes, respectively; that is, the values that the estimators Eqs. 7 and 8 take with the sample answers of the customers and providers:

9 $$ \begin{array}{@{}rcl@{}} \mathbf{\widehat{{x}}}_{j}&= & \left( f(u_{11j}, \cdots,u_{{n_{x_{j}}}1j}), \dots, f(u_{1mj}, \cdots,u_{{n_{x_{j}}}mj}) \right)\\ &=&\left( {\widehat{x}}_{1j}, \cdots, {\widehat{x}}_{mj} \right); \ j=1, \cdots, L \end{array} $$

10 $$ \begin{array}{@{}rcl@{}} \mathbf{\widehat{{y}}}_{j} &= & \left( g(w_{11j}, \cdots,w_{{n_{y_{j}}}1j}), \dots, g(w_{1sj}, \cdots,w_{{n_{y_{j}}}sj}) \right)\\ &=&\left( {\widehat{y}}_{1j}, \cdots, {\widehat{y}}_{sj} \right); \ j=1, \cdots, L \end{array} $$

Using ${\left ({\widehat {{\mathbf X}}}_{\mathbf j}, \ {\widehat {{\mathbf Y}}}_{\mathbf j}\right )}_{j=1,{\dots } ,L}$ in the Table 1 models, we obtain the estimators $ {\left ({{\widehat {\varphi } }_{j}}\right )}_{j=1,\dots ,L} $ of the population efficiency scores $ {\left ({{\varphi }_{j}}\right )}_{j=1,\dots ,L} $, on the understanding that the DEA model is maximized, or minimized, with the data ${\left ({\widehat {{\mathbf x}}}_{\mathbf j}, \ {\widehat {{\mathbf y}}}_{\mathbf j}\right )}_{j=1,{\dots } ,L}$ in order to obtain the estimation $ {{\widehat {\omega } }_{j}}$ of the estimator $ {{\widehat {\varphi } }_{j}}$.

Therefore, our statistical model $ \left ({\Omega }, P \right ) $ corresponds to independent, random samples in each SPU, that is, the sample space is ${\Omega }={\prod }_{j=1}^{L}{\Omega }_{j}$, where $ {\Omega }_{j}=\left \{ \text {samples} \mathbf {u_{kj}} \text {of size} n_{x_{j}} \text { and} \text { samples} \mathbf {w_{hj}} \text {of size} n_{y_{j}}\right .$ $\left . \text {in SPU}_{j} \right \rbrace $, and the probability *P* depends on the sample design used.

The first objective of this paper, having fixed *δ* ∈ (0,1) and $\alpha \in \left (0,1\right )$, is to obtain the provider and customer sampling size, $N_{x_{j}}$ and $N_{y_{j}}$, respectively, to estimate ${\widehat {X}}_{j} $ and ${\widehat {Y}}_{j} $ and the estimator ${\widehat {\varphi }}_{j} $ such that:

11 $$ P\left( \left\vert{\widehat{\varphi}}_{j}-{\varphi}_{j}\right\vert\leq\delta\right)\geq 1-\alpha;\ j=1,\dots,\ L. $$

The second objective is to determine a confidence interval for the populational efficiency, in each SPU, with a fixed accuracy.

## How many providers and customers need to be interviewed?

The provider and customer sample size problem Eq. 11 can only be analytically resolved in the case of one input and one output and the CCR model. A rigorous proof of all the results are provided in the Appendix I.

### CCR model with one provider and one customer opinion index

Let us consider these assumptions:

1. Fixed *L* SPUs.
2. One provider population opinion index $\left \lbrace X_{j}\right \rbrace _{j=1,{\dots } ,L}$ as input and one customer population opinion index $\left \lbrace Y_{j}\right \rbrace _{j=1,{\dots } ,L}$ as output. We consider $\left \lbrace {\widehat {X}}_{j}\right \rbrace _{j=1,{\dots } ,L}$ and $\left \lbrace {\widehat {Y}}_{j}\right \rbrace _{j=1,{\dots } ,L}$ to be the corresponding estimators.
3. CCR model Eq. 1 with output orientation (CCR-O).

Let ${(Z_{j}=\frac {Y_{j}}{X_{j}})}_{j=1,\ {\dots } ,\ L}$ and ${({\widehat {Z}}_{j}=\frac {{\widehat {Y}}_{j}}{{\widehat X}_{j}})}_{j=1,{\dots } ,L}$. In this situation and with the given notation, the population efficiency obtained with the CCR-O model and its estimator are:

12 $$ \begin{array}{@{}rcl@{}} && {\varphi }_{j}=\frac{Z_{j}}{{max}_{j=1,{\dots} ,\ L}\left\{Z_{j}\right\}} \end{array} $$

13 $$ \begin{array}{@{}rcl@{}} && {\widehat{\varphi }}_{j}=\frac{{\widehat{Z}}_{j}}{{max}_{j=1,\dots,L}\left\{{\widehat{Z}}_{j}\right\}}. \end{array} $$

Having fixed *p* ∈ (0,1), we thus consider the sets of Ω:

14 $$ \begin{array}{@{}rcl@{}} & A_{j}=\left( \left|{\widehat{Y}}_{j}-Y_{j}\right|\le pY_{j}{\mathbf \ } \right) \cap \left( \left|{\widehat{X}}_{j}-X_{j}\right|\le pX_{j} \right);\ j=1,\ {\dots} ,\ L\ \end{array} $$

The following Lemmas 1, 2 and 3 are used to prove Theorem 1 which, in this particular case, establishes the relation between the accuracy of the provider and customer opinion estimation indexes and that of the population CCR-O efficiency estimation.

#### **Lemma 1**

Under assumptions C1, C2 and C3, with the given notation, fixed *p* ∈ (0,1) and *m* such that $ Z_{m}={max}_{j=1,{\dots } ,\ L}\left \{Z_{j}\right \}$, then:

$$ \begin{array}{@{}rcl@{}} A_{j}&=&\left( \frac{(1-p)}{(1+p)}{\widehat{Z}}_{j}\le Z_{j}\le \frac{(1+p)}{(1-p)}{\widehat{Z}}_{j}\right)\\ &=&\left( Z_{j}\frac{(1-p)}{(1+p)}\le {\widehat{Z}}_{j}\le Z_{j}\frac{(1+p)}{(1-p)}\right)\\ &=&\left( \frac{{\widehat{Z}}_{j} (1-p)}{Z_{m}\left( 1+p\right)}\le {\varphi }_{j}\le \frac{{\widehat{Z}}_{j}(1+p)}{Z_{m}\left( 1-p\right)}{\mathbf \ }\right). \end{array} $$

#### **Lemma 2**

Under assumptions C1, C2 and C3, with the given notation, fixed *p* ∈ (0,1), *m* and *Z*_*m*_ defined as in Lemma 1 and *l* such that :

$${\widehat{Z}}_{l}={max}_{j=1,\dots,L}\left\{{\widehat{Z}}_{j}\right\}$$

Consider the set of Ω

15 $$ \begin{array}{@{}rcl@{}} & B=\left( Z_{m}\frac{\left( 1-p\right)}{\left( 1+p\right)}\le {\widehat{Z}}_{l}\le Z_{m}\frac{(1+p)}{(1-p)}\right), \end{array} $$

then:

*m* = *l* implies *A*_*l*_ = *B*

*m*≠*l* implies *A*_*l*_ ∩ *A*_*m*_ ⊂ *B*

#### **Lemma 3**

Under assumptions C1, C2 and C3, with the given notation, fixed *p* ∈ (0,1) and for any $ j=1,\ {\dots } ,\ L$ we have that

$$ \begin{array}{@{}rcl@{}} A_{j}\cap B&\subset& \left\{{\widehat{\varphi }}_{j}\frac{(1-p)^{2}}{(1+p)^{2}}\le \frac{{\widehat{Z}}_{j}(1-p)}{Z_{m}\left( 1+p\right)}\le {\varphi }_{j}\right\}\\ &\cap& \left\{{\varphi }_{j}\le \frac{{\widehat{Z}}_{j}(1+p)}{Z_{m}\left( 1-p\right)}\le {\widehat{\varphi }}_{j}\frac{(1+p)^{2}}{(1-p)^{2}}\right\} \end{array} $$

#### **Theorem 1**

Let us consider the assumptions C1, C2 and C3. Having fixed *p* ∈ (0,1) and $\alpha \in \left (0,1\right )$, if $\ P\left (A_{j}\right )\ge 1-\alpha \ \ \forall j=1,{\dots } ,L$, then

16 $$ P\left( {\widehat{\varphi }}_{j}\frac{(1-p)^{2}}{\left( 1+p\right)^{2}}\le \varphi_{j} \le {\widehat{\varphi }}_{j}\frac{\left( 1+p\right)^{2}}{\left( 1-p\right)^{2}} \right)\ge {\left( 1-\alpha \right)}^{3}. $$

Lemma 4 is an instrumental result, used to prove Theorem 2 and Corollary 1, which establishes how to determine the provider and customer sample size in each SPU, so the CCR-O efficiency estimator has the precision fixed in Eq. 11.

#### **Lemma 4**

Under the hypotheses of Theorem 1, if

$$ {\varphi{}}_{j}\in{}\left[{\widehat{\varphi}}_{j}\frac{\left( 1-p\right)^{2}}{(1+p)^{2}},min\left\{1,{\widehat{\varphi}}_{j}\frac{(1+p)^{2}}{(1-p)^{2}}\right\}\right] $$

then

$$ {\varphi{}}_{j}\in{}\left[{\widehat{\varphi{}}}_{j}-\frac{4p}{(1+p)^{2}},\ min\left\{1,{\widehat{\varphi{}}}_{j}+\frac{4p}{(1+p)^{2}}\ \right\}\right] $$

#### **Theorem 2**

Let us consider the assumptions C1, C2 and C3. Having fixed *p* ∈ (0,1) and $\alpha \in \left (0,1\right )$, for every $j=1,\ \dots , \ L$, let $N_{x_{j}}$ be the sampling size in the SPU_*j*_, such that

17 $$ P\left( \left\vert{\widehat{X}}_{j}-X_{j}\right\vert{}\leq p X_{j}\right)\geq{}\sqrt[6]{1-\alpha{}} $$

and $N_{y_{j}}$ be the sampling size such that

18 $$ P\left( \left\vert{\widehat{Y}}_{j}-Y_{j}\right\vert{}\leq {p}Y_{j}\right)\geq{}\sqrt[6]{1-\alpha} $$

then

19 $$ P\left( \left\vert{}{\widehat{\varphi{}}}_{j}-{\varphi{}}_{j}\right\vert{}\leq \frac{4p}{(1+p)^{2}}\right)\geq{}1-\alpha{};\ \forall j=1,\dots,\ L. $$

#### **Corollary 1**

Let us consider the assumptions C1, C2 and C3. Having fixed *δ* ∈ (0,1) and $\alpha \in \left (0,1\right )$, for every $j=1,\dots ,L$, let $N_{x_{j}}$ be the sampling size in the SPU_*j*_, such that

20 $$ P\left( \left\vert{}{\widehat{X}}_{j}-X_{j}\right\vert{}\leq \left( \frac{2-\delta -2 \sqrt{1-\delta}}{\delta} \right) X_{j}\right)\geq{}\sqrt[6]{1-\alpha{}} $$

and $N_{y_{j}}$ be the sampling size such that

21 $$ P\left( \left\vert{}{\widehat{Y}}_{j}-Y_{j}\right\vert{}\leq \left( \frac{2-\delta -2 \sqrt{1-\delta}}{\delta} \right) Y_{j}\right)\geq{}\sqrt[6]{1-\alpha} $$

then

22 $$ P\left( \left\vert{}{\widehat{\varphi{}}}_{j}-{\varphi{}}_{j}\right\vert{}\leq \delta\right)\geq{}1-\alpha{};\ \forall{}j=1,\dots,\ L $$

Remark 1 gives the explicit formula to obtain the sample size under the usual simple random sample without replacement sample design.

#### *Remark 1*

If the design in each SPU is simple random sampling without replacement, and the output (i.e. input) is the mean of all the answers of the population to a survey item, then the sampling size $n_{\theta _{j}}$ that it verifies

$$\ P\left( \left|{\widehat{\theta}}_{j}-\theta_{j}\right|\le p\theta_{j}\right)\ge 1-{\alpha}_{1}; \ \theta=X  \text{or}  Y; \ p, {\alpha}_{1} \in \left( 0,1\right) $$

is ([36])

23 $$ n_{\theta_{j}}\ge \frac{n_{oj}}{\left( \frac{n_{oj}}{N_{j}}+1\right)} $$

with $n_{oj}=\frac {{\tau }^{2}_{1-{{\alpha }_{1} }/{2}}}{{\left (p\theta _{j}\right )}^{2}}{{\sigma }_{\theta _{j}}}^{2}$ and ${\tau }_{1-{\alpha _{1} }/{2}}={\phi }^{-1}\left (1-{\alpha _{1} }/{2}\right )$, where ${{\sigma }_{y_{j}}}^{2}$ is the population variance and *ϕ* the normal standard distribution function.

### CCR or BCC model with two or more provider and/or customer opinion indexes

In this section, we report on our simulation study to check that Theorem 2 and Corollary 1 also work in the BCC model with two or more estimated provider and/or customer opinion indexes.

If we consider *m* items (the same in all SPUs) to estimate the *m* provider opinion indexes (inputs) $\left (X_{1j},\ {\dots } ,\ X_{mj}\right )$ with $\left ({\widehat {X}}_{1j}, \dots , {\widehat {X}}_{mj}\right ) $, Remark 1 calculates the sample size $n_{x_{ij}}$ necessary to achieve

24 $$ P\left( \left\vert{\widehat{X}}_{ij}-X_{ij}\right\vert\leq p X_{ij}\right)\geq{}\sqrt[6]{1-\alpha{}}; \ i=1,..., m; \ j=1, ..., L. $$

We propose to determine the provider sample size $N_{x_{j}}$ in the SPU_*j*_ as

25 $$ n_{x_{j}}= max_{i=1, ..., m}\left\lbrace n_{x_{ij}}\right\rbrace, $$

i.e., if we consider *s* items (the same in all SPUs) to estimate the *s* customer opinion indexes (outputs) the provider sample size $N_{y_{j}}$ in the SPU_*j*_ is determined as

26 $$ n_{y_{j}}= max_{r=1, ..., s}\left\lbrace n_{y_{rj}}\right\rbrace, $$

where $n_{y_{rj}}$ is the sample size necessary to achieve

27 $$ P\left( \left\vert{\widehat{Y}}_{rj}-Y_{rj}\right\vert\leq p Y_{rj}\right)\geq{}\sqrt[6]{1-\alpha{}}; \ r=1,..., s; \ j=1, ..., L. $$

#### Simulation study

We use the [13] health center data (Table 2) to simulate a population model: in the jth health center a population size of providers and customers, $N_{x_{j}} $ and $N_{y_{j}}$, are generated from a random uniform distribution, according to the intervals $ \left [10000, 50000 \right ] $ and $ \left [30000, 80000 \right ] $, respectively. For each provider, we generate two item answers $ \left (U_{k1j}, U_{k2j} \right ) $, i.e., for each customer $ \left (W_{h1j}, W_{h2j} \right ) $, from a bivariate normal distribution as

$$\left( \begin{array}{c} U_{k1j}\\ U_{k2j} \end{array}\right) \hookrightarrow N_{2}\left( \left( \begin{array}{c} doct_{j}\\ nurs_{j} \end{array}\right) , \left( \begin{array}{cc} doc{t_{j}^{2}}/4 & 0 \\ 0 & nur{s_{j}^{2}}/4 \end{array}\right)\right); k=1, ...., N_{x_{j}}; j=1, ..., 12 $$

$$ \left( \begin{array}{c} W_{h1j}\\ W_{h2j} \end{array}\right) \hookrightarrow N_{2}\left( \left( \begin{array}{c} out_{j}\\ inp_{j} \end{array}\right) , \left( \begin{array}{cc} ou{t_{j}^{2}}/4 & 0\\ 0 & in{p_{j}^{2}}/4 \end{array}\right)\right); h=1, ...., N_{y_{j}}; j=1, ..., 12 $$

where *doc*_*j*_, *nurs*_*j*_ and *out*_*j*_, *inp*_*j*_ are the original value doctor, nurse and outpatient, inpatient of the jth health center, columns 2, 3, 4 and 5 of Table 2, respectively. We consider the population mean to the simulated answers to the provider and customer items in the jth center, *j* = 1,...,12, to simulate the population inputs and outputs, columns 3, 4, 5 and 6 in Table 3:

$$ \begin{array}{@{}rcl@{}} &&\left( X_{1j}, X_{2j}\right) =\left( \frac{{\sum}_{k=1}^{N_{x_{j}}}{{U}}_{k1j}}{N_{x_{j}}}, \frac{{\sum}_{k=1}^{N_{x_{j}}}{{U}}_{k2j}}{N_{x_{j}}}\right)\\ &&\left( Y_{1j}, Y_{2j} \right) =\left( \frac{{\sum}_{h=1}^{N_{y_{j}}}{{W}}_{h1j}}{N_{y_{j}}}, \frac{{\sum}_{h=1}^{N_{y_{j}}}{{W}}_{h2j}}{N_{y_{j}}} \right) \end{array} $$

**Table 2:** Number of doctors, nurses, outpatients and inpatients in 12 health centers

Health center	Doctor	Nurse	Outpatient	Inpatient	Efficiency score CCR-O	Efficiency score BCC-O
1	2.0	15.1	10	9	1	1
2	1.9	13.1	15	5	1	1
3	2.5	16	16	5.5	0.883	0.925
4	2.7	16.8	18	7.2	1	1
5	2.2	15.8	9.4	6.6	0.763	0.767
6	5.5	25.5	23	9	0.835	0.955
7	3.3	23.5	22	8.8	0.902	1
8	3.1	20.6	15.2	8	0.796	0.826
9	3	24.4	19	10	0.960	0.990
10	5	26.8	25	10	0.871	1
11	5.3	30.6	26	14.7	0.955	1
12	3.8	28.4	25	12	0.958	1
*Font: Table 1.5* [13]

**Table 3:** Simulated population model

Health center	Provider population size $\mathbf {N_{x_{j}}}$	X1	X2	Customer Population size $\mathbf {N_{y_{j}}}$	Y1	Y2	Population efficiency score CCR-O	Population efficiency score BCC-O
1	16684	2.04	15.52	57128	10.29	9.25	1	1
2	26950	1.95	13.40	36283	15.36	5.15	1	1
3	26583	2.56	16.41	65876	16.41	5.66	0.882	0.926
4	18974	2.77	17.22	33569	18.44	7.37	1	1
5	43106	2.26	16.26	62522	9.63	6.79	0.761	0.764
6	21305	5.65	26.27	68349	23.68	9.24	0.834	0.952
7	10599	3.40	23.98	36417	22.61	9.07	0.901	1
8	27432	3.18	21.13	73596	15.60	8.22	0.798	0.828
9	11113	3.09	24.94	38962	19.54	10.29	0.954	0.987
10	31717	5.15	27.44	76479	25.70	10.28	0.875	1
11	39802	5.44	31.44	36356	26.63	15.12	0.955	1
12	32282	3.91	29.26	55847	25.67	12.33	0.955	1

The last two columns in Table 3 show the population efficiency scores CCR and BCC with output orientation.

To check the relation between sample size, estimation of the input/output indexes and estimation of the DEA efficiency, using Theorem 2 and Corollary 1 and these simulated population data, we follow the next steps:

i. In the jth health center, $ j=1,\dots ,12 $, a previous simple random sample without replacement of 25 providers $\left (n_{x_{j}}^{(0)}=25 \right ) $ is taken to estimate the two inputs $ \left ({\widehat {x }}_{1j}^{(0)}, {\widehat {x }}_{2j}^{(0)} \right ) $, and their variances $\left ({\widehat {\sigma }}_{1jx}^{2(0)}, {\widehat {\sigma }}_{2jx}^{2(0)}\right ) $ using the sample means and the sample quasi-variances, respectively, i.e., we estimate the two outputs $\left ({\widehat {y }}_{1j}^{(0)}, {\widehat {y }}_{2j}^{(0)} \right ) $ and their variances, $ \left ({\widehat {\sigma }}_{1jy}^{2(0)}, {\widehat {\sigma }}_{2jy}^{2(0)} \right ) $ with a previous simple random sample without replacement of 25 customers $ \left (n_{y_{j}}^{(0)}=25 \right ) $.
ii. Fixed *δ* = 0.1 or 0.2 and 1 − *α* = 0.9 as in Corollary 1, and with the estimates of step i., the sample sizes, $N_{x_{j}}$ and $N_{y_{j}}$, are determined using Eqs. 23, 25 and 26.
iii. In the jth health center, the simple random samples without replacement of size $N_{x_{j}}$ and $ n_{y_{j}} $ are taken and the inputs $\left ({\widehat {x }}_{1j} ,{\widehat {x }}_{2j}\right ) $ and outputs $\left ({\widehat {y }}_{1j} ,{\widehat {y}}_{2j}\right ) $ are estimated. With the data $\left \lbrace {\left ({\widehat {x }}_{1j} ,{\widehat {x }}_{2j},{\widehat {y }}_{1j} ,{\widehat {y}}_{2j}\right ) }\right \rbrace _{j=1,...,12}$, the estimated efficiencies ${\left \lbrace {\widehat {\omega }}_{j}\right \rbrace }_{j=1,...,12}$ are obtained, maximizing the LP model Eqs. 3 or 4 with output orientation.
iv. One thousand iterations of step iii. are carried out obtaining, for the jth health center, 1000 estimated efficiency scores $ {\left \lbrace {{\widehat {\omega }}_{j}^{(k)}}\right \rbrace }_{k=1, ..., 1000} $ and 1000 intervals
  28 $$ H_{j}^{(k)}=\left[{\widehat{\omega }}_{j}^{(k)}-\delta,min\left\{{\widehat{\omega }}_{j}^{(k)}+\delta,1\right\}\right]; \ k=1,{\dots} ,1000 $$
v. The probability $P\left (\left \vert {}{\widehat {\varphi {}}}_{j}-{\varphi {}}_{j}\right \vert {}\leq \delta \right ) $ is approximated by calculating
  29 $$ C_{j}=\frac{1}{1000}{\sum}^{1000}_{k=1}{I_{\left( {\varphi }_{j}\in H_{j}^{(k)}\right)}} $$

Table 4 shows the sampling sizes, $N_{x_{j}}$ and $n_{y_{j}} $, obtained for the jth health center in the last iteration, for the two values of *δ* and *α* = 0.1. In health center 3 or 6, the customer sample size increases up to 6 times when fixing a maximum *δ* = 0.2 to 0.1.

The probabilities $P\left (\left \vert {}{\widehat {\varphi {}}}_{j}-{\varphi {}}_{j}\right \vert {}\leq {}\delta {}\right ) $ approximated with Eq. 29 take the value one for all the health centers, the two values of *δ*, the CCR-O or BCC-O model and with *α* = 0.1. Therefore, the confidence intervals for the population efficiency score *φ*_*j*_, obtained with the samples of the size of Table 4 and Corollary 1, are very conservative. However, in the next section, we will see that these same sample sizes allow less conservative bootstrap DEA efficiency confidence intervals to be obtained.

**Table 4:** Customer and provider sample size, taking two values of *δ*, 0.2 or 0.1, and a confidence 1 − *α* = 0.9

	*δ* = 0.2		*δ* = 0.1	
Health center	Provider sample size	Customer sample size	Provider sample size	Customer sample size
1	463	560	1747	2654
2	681	358	1858	3627
3	398	380	2481	1268
4	544	487	1047	1749
5	495	404	2440	1807
6	441	403	1650	2397
7	399	551	1533	2131
8	492	418	2010	2519
9	542	371	1495	2045
10	394	515	1731	2900
11	457	612	2872	1456
12	464	257	1265	2015

## Description of the bootstrap efficiency confidence interval technology

Bootstrap uses resampling to estimate the value of a parameter of a population ([18]). For the problem suggested in Section 2, we propose bootstrap resampling of the samples of the provider and customer answers to the opinion item to obtain confidence intervals for the population efficiencies, following these steps:

i. Having fixed *δ* and a probability 1 − *α*, we determine the sample sizes $N_{x_{j}}$ and $N_{y_{j}}$, in the SPU_*j*_, using Corollary 1, Remark 1 and Eqs. 25 and 26.
1. In the SPU_*j*_, we take a provider and a customer simple random samples without replacement $ \left \lbrace \mathbf {u_{kj}}= \left (u_{k1j}, ..., u_{kmj} \right )\right \rbrace _{k=1, ..., n_{x_{j}}} $ and $ \left \lbrace \mathbf {w_{hj}}= \left (w_{h1j}, ..., w_{hsj} \right )\right \rbrace _{h=1, ..., n_{y_{j}}} $, respectively, to estimate the provider and customer opinion indexes $ \left ({\widehat {{x}}}_{1j},\dots ,{\widehat {{x}}}_{mj} \right ) $ and $ \left ({\widehat {{y}}}_{1j},\dots ,{\widehat {{y}}}_{sj} \right ) $, respectively, for example, with the sample means:
  30 $$ \begin{array}{@{}rcl@{}} && \widehat x_{ij}=\frac{{\sum}_{k=1}^{n_{x_{j}}} u_{kij}}{n_{x_{j}}};  i=1, ..., m;  j=1, ...L \end{array} $$
  31 $$ \begin{array}{@{}rcl@{}} && \widehat y_{rj}=\frac{{\sum}_{h=1}^{n_{y_{j}}} w_{hrj}}{n_{y_{j}}};  r=1, ..., s;  j=1, ...L. \end{array} $$
ii. In the SPU_*j*_, we take a bootstrap sample with replacement $ \left \lbrace \mathbf {u_{kj}}^{*}\right \rbrace _{k=1, ..., n_{x_{j}}} $ from $ \left \lbrace \mathbf {u_{kj}}\right \rbrace _{k=1, ..., n_{x_{j}}} $, i.e., $ \left \lbrace \mathbf {w_{hj}}^{*}\right \rbrace _{h=1, ..., n_{y_{j}}} $ of size $n_{y_{j}} $ from $ \left \lbrace \mathbf {w_{hj}}\right \rbrace _{h=1, ..., n_{y_{j}}} $, with which we obtain the bootstrap version of the *m* inputs, $\mathbf {{\widehat {x}}_{j}^{*}}=\left (\widehat {x}_{1j}^{*}, ..., \widehat {x}_{mj}^{*}\right )$, and *s* outputs, $\mathbf {{\widehat {y}}_{j}^{*}}=\left (\widehat {y}_{1j}^{*}, ..., \widehat {y}_{sj}^{*}\right )$. With the data $ {\left \lbrace \left (\widehat {x}_{1j}^{*}, ..., \widehat {x}_{mj}^{*},\widehat {y}_{1j}^{*}, ..., \widehat {y}_{sj}^{*}\right )\right \rbrace }_{j=1,{\dots } ,L}$ and the DEA model of Table 1, we obtain the bootstrap version of the estimated DEA efficiency, $ {\left ({\widehat {\omega }^{*}}_{j}\right ) }_{j=1,...,L} $.
iii. The step iii. is repeated *B* times and the *B* bootstrap versions of the estimated DEA efficiency for the SPU_*j*_, *j* = 1,...,*L*, are ${\left \{{\widehat {\omega }}^{*(b)}_{j}\right \}}_{b\text {=1,{\dots } ,\ }\ B}$.
2. In the SPU_*j*_, the observed percentile bootstrap confidence interval for the population efficiency score *φ*_*j*_ is obtained, having fixed a coverage intention of level $\text {1-}\alpha ^{\prime }$, as
  32 $$ I_{j}=\left[ {\widehat{\omega}}^{*(\alpha^{\prime}/2)}_{j},{\widehat{\omega}}^{*(1-\alpha^{\prime}/2)}_{j}\right] $$
  where ${\widehat {\omega }}^{*(\alpha )}_{j}$ is the *α*-percentile of the *B* values ${\left \{{\widehat {\omega }}^{*(b)}_{j}\right \}}_{b\text {=1,{\dots } ,\ }\ B}$.

### Simulation study

To illustrate the bootstrap efficiency confidence interval methodology and to check the estimation quality of the DEA population efficiency obtained, a simulation was performed using the population model simulated from Table 3.

First, we fixed *δ* = 0.2 and the confidence 1 − *α* = 0.9 to calculate the provider and customer sample size to estimate the input and output data; supposing a simple random sample without replacement, these sample sizes are columns 2 and 3 in Table 4.

The steps ii.-v. are iterated 1000 times, fixing the confidence of the bootstrap efficiency interval $1-\alpha ^{\prime }=0.9 \ \text {or} \ 0.95$ and 2000 resampling Bootstraps (*B* = 2000).

The confidence of the bootstrap interval for the DEA efficiency in the SPU_*j*_ is approximated with:

33 $$ C_{j}\mathrm{=}\frac{\mathrm{1}}{\text{1000}}\sum\limits^{\text{1000}}_{k\text{=1}}{I_{\left( \varphi_{j}\in {I_{j}}^{\left( k\right)}\right)}};  j=1, ..., 12 $$

where $ {\left \{{I_{j}}^{(k)}=[{I_{j}}^{\left (k\right )L},\ {I_{j}}^{(k)U}]\right \}}_{k=1, ..., 1000} $ are the 1000 bootstrap efficiency confidence intervals obtained in step v.

Table 5 shows the approximate confidence of the bootstrap intervals, output orientation DEA models. We observe that the control of the coverage level $1-\alpha ^{\prime }$ leads to the achievement of the confidence of the bootstrap efficiency interval required by the experimenter.

**Table 5:** Simulated confidence of bootstrap efficiency intervals, having fixed *δ* = 0.2

	$ 1-\alpha ^{\prime }=0.90 $		$ 1-\alpha ^{\prime }=0.95 $	
SPU	CCR-O	BCC-O	CCR-O	BCC-O
**1**	100	100	100	100
**2**	100	100	100	100
**3**	92.5	91.9	96.5	95.8
**4**	98.4	99.9	99.5	99.9
**5**	92.2	91	96.5	95.5
**6**	89.7	89.9	95.2	94.6
**7**	89.3	98.3	93.7	99.2
**8**	91.1	92.4	96.3	97.3
**9**	91.9	91.4	96.4	95.8
**10**	90.6	98.9	94.8	99.4
**11**	89.4	100	95	100
**12**	92	100	95	100

Output-oriented CCR and BCC model

The amplitude of the bootstrap efficiency confidence intervals is analysed with the approximation of the expected value of the bounds

34 $$ E\left( {I_{j}}^{L}\right)=\frac{{\sum}^{\text{1000}}_{k\text{=1}}{{I_{j}}^{\left( k\right)L}}}{1000} ~\text{and}~ E\left( {I_{j}}^{U}\right)=\frac{{\sum}^{\text{1000}}_{k\text{=1}}{{I_{j}}^{\left( k\right)U}}}{1000}. $$

Table 6 shows the approximation of the expected values of the bounds of the bootstrap efficiency confidence interval, considering the BCC model with output orientation. If we look at the SPUs in which the expected efficiency confidence interval contains the one, {1, 2, 4, 7, 10, 11, 12}, these SPUs coincide with the efficient population units (value 1 in column 9 from Table 3). As expected, the increase in the trust $1-\alpha ^{\prime } $ of the interval Bootstrap leads to an increase in the amplitude.

**Table 6:** Approximation of the expected values of the bounds of the bootstrap efficiency confidence intervals, *δ* = 0.2, *α*= 0.1 and $\left (1-\alpha ^{\prime }\right )=0.9 \text {or} 0.95$

	$ 1-\alpha ^{\prime }=0.9 $		$ 1-\alpha ^{\prime }=0.95$	
SPU	${E}\left ({{{I}}_{{i}}}^{{L}}\right )$	${E}\left ({{{I}}_{{i}}}^{{U}}\right )$	${E}\left ({{{I}}_{{i}}}^{{L}}\right )$	${E}\left ({{{I}}_{{i}}}^{{U}}\right )$
1	1	1	1	1
2	1	1	1	1
3	0.880	0.966	0.872	0.974
4	0.979	1	0.972	1
5	0.737	0.821	0.731	0.835
6	0.894	0.985	0.885	0.990
7	0.956	1	0.948	1
8	0.796	0.854	0.791	0.860
9	0.941	0.998	0.932	0.999
10	0.954	1	0.943	1
11	1	1	1	1
12	1	1	1	1

Output-oriented BCC model

In conclusion, the bootstrap efficiency confidence interval methodology has the advantage that, after determining the provider and customer sample size using Corollary 1, Remark 1 and Eqs. 25 and 26, the experimenter can achieve the confidence required, $1-\alpha ^{\prime }$, to estimate the population efficiency.

## Application to the Spanish health system

This section provides an empirical analysis of health production for Spain’s 18 Autonomous Communities (CCAA).

Spain’s Health Ministry has, for some time, been compiling the statistic ”Health Barometer” (HB), where a group of individuals is selected in each CCAA, and a questionnaire is carried out to test the health system. One of the survey question blocks take the opinion of the individual concerning the attention provided by the doctors of primary attention and pediatrics in Spain with the following items:

Either from your personal experience or in your own opinion, we would like you to evaluate the following aspects of the public health service, concerning the attention provided by the GP or the paediatrician. Do so using the scale of 1 to 10, where 1 means ’totally unsatisfactory’ and 10 means ’totally satisfactory’.

- **(P-1)** The attention received from the healthcare personnel
- **(P-2)** The time dedicated by the doctor to each patient
- **(P-3)** The confidence and security that the doctor transmits
- **(P-4)** The information received concerning your health problem
- **(P-5)** The time between making the appointment and the visit to the doctor.

A principal components analysis (PCA) is carried out over these 5 items.The PCA is a statistical technique for reducing the variable dimensionality of the dataset, minimizing information loss. It does so by creating new uncorrelated variables that successively maximize the explained variance of the dataset ([24]). The first component (PCA1) obtained, interpretable as the size of satisfaction, explains 98.1% of the variability. The sample mean of the answers to this component in every CCAA is our estimated output index, interpreted as the patient mean satisfaction with the CCAA’s healthcare system (column 9 in Table 7).

**Table 7:** Spain’s Autonomous Communities, population size and sample size of providers, estimation of the inputs, population size and sample size of customers and estimation of the output

			Input data			Output data		
CCAA	$ {N_{x_{j}}} $	$ {n_{x_{j}}}$	$ {\overline {C1}} $	$ {\overline {C2}} $	${\overline {C3}} $	$ {N_{y_{j}}} $	$ {N_{y_{j}}} $	$ {\overline {PCA1}} $
**1**	5960	950	0.361	0.420	27.34	8399043	726	15.830
**2**	1132	76	0.947	0.092	31.91	1317847	319	18.109
**3**	762	133	0.699	0.263	27.73	1051229	306	16.632
**4**	666	107	0.907	0.224	31.66	1104479	286	16.634
**5**	1486	32	0.594	0.219	35.16	2100306	349	16.462
**6**	444	140	0.793	0.157	22.86	585179	243	17.610
**7**	1600	203	0.419	0.320	25.43	2059191	348	16.735
**8**	2630	383	0.705	0.272	29.21	2472052	382	16.999
**9**	5441	243	0.934	0.243	29.37	7508106	707	15.947
**10**	3553	290	0.645	0.283	27.67	4980689	540	16.250
**11**	950	178	0.562	0.124	25.00	1092997	315	16.202
**12**	2194	136	0.765	0.191	28.13	2732347	405	15.965
**13**	4383	431	0.594	0.179	30.08	6436996	607	16.379
**14**	1072	197	0.457	0.051	21.89	1467288	320	16.649
**15**	497	150	0.940	0.060	21.00	640476	269	17.657
**16**	1780	291	0.959	0.107	22.12	2189257	384	16.650
**17**	257	108	0.759	0.157	28.24	317053	241	17159
**18**	93	12	0.333	0.083	20.83	169847	464	14.707

The input data is estimated using the results of the “Survey on the current situation of GPs in Spain”, carried out by the Spanish Medical Colleges Organization (OMC) in 2015 on the population of Spanish GPs and paediatricians. We use the following items:

- **(C1)** Workload as number of patients attended per day, answering 1 if the workload is normal or low (inferior to 40 patients) and 0 if the workload is high.
- **(C2)** Occupation of the team of doctors, answering 1 if the occupation is normal or low and 0 if it is high.
- **(C3)** Time dedicated to ongoing training.

The sample means of the doctors, answers to these items in each CCAA estimate the three opinion indexes used as inputs: the proportion of doctors with a normal or low workload $\left (\mathbf {\overline {C1}} \right ) $, the proportion of teams of doctors with a normal or low occupation $\left (\mathbf {\overline {C2}} \right ) $, and the mean time doctors dedicated to ongoing training $\left (\mathbf {\overline {C3}} \right ) $. From our point of view, an increase in the value of these input indexes in the population of doctors of a CCAA would lead to a bigger satisfaction in the population of patients attended in the same CCAA. Columns 2 and 3 of Table 7 show the population and sample size of doctors (i.e., columns 7 and 8 of patients).

Table 8 shows the results of the estimation point $\left ({\widehat {{\mathbf \omega }}}_{{\mathbf i}}\right )$ and bootstrap interval of the population efficiency scores, with confidence $ 1-\alpha ^{\prime }=0.9 $, in each of Spain’s CCAAs, considering variable returns-to-scale and output orientation. The CCAAs in which the hypothesis of an efficient public health service (DEA efficiency equal one) is rejected are {3, 4, 5, 8, 9 10, 11, 12, 13, 17}. The CCAAs which are benchmark for the rest are {2 6, 7, 14 , 15}, because the upper and lower confidence interval bounds have value one. In general, the efficiency in all CCAAs is good, the inferior bound of the confidence interval is superior to 0.9 in all the cases, except in the CCAA {12}, according to our results, the CCAA with the least efficient health service.

**Table 8:** Spain’s CCAAs, public health efficiency scores estimated point and by bootstrapping confidence interval, with $ 1-\alpha ^{\prime }=0.9 $, using data from The Health Barometer of 2015 and the ”Survey on the situation of Primary Care doctors in Spain, 2015”

		Bootstrap efficiency confidence interval	
CCAA	${\widehat {{\omega }}}_{{i}}$	Lower bound	Upper bound
1	1	0.941	1
2	1	1	1
3	0.956	0.923	0.977
4	0.925	0.903	0.950
5	0.961	0.924	0.994
6	1	0.983	1
7	1	0.988	1
8	0.977	0.949	0.989
9	0.886	0.868	0.905
10	0.941	0.915	0.955
11	0.954	0.928	0.984
12	0.910	0.883	0.926
13	0.958	0.933	0.972
14	1	1	1
15	1	1	1
16	0.941	0.918	1
17	0.979	0.949	0.998
18	1	0.876	1

BCC-O model

## Conclusions

The approach presented in this paper provides a step towards producing valid estimates of technical efficiency in public services, using provider and customer satisfaction-opinion indexes estimated with samples. These indexes measure the service quality from the perspective of both the provider and the customer.

We have developed statistical results for comparing the efficiencies of public services. These results are novel in the sense that: (i) We resolve the problem of determining the customer and provider sample size necessary to estimate the opinion indexes and the population efficiency with an accuracy fixed a priori; (ii) We build confidence intervals for the population DEA efficiency using bootstrap replicates of the providers sample and the customers sample in each public service; (iii) It is possible to achieve the level of confidence of the bootstrap efficiency confidence interval required by the experimenter (iv) The DEA models used are the original linear programming models; (iv) The new approach can be readily implemented. As far as we know, the approach of this paper has not been attempted in the literature.

While this study provides a useful methodology to measure public service efficiency, its limitations should also be acknowledged. First, the results can only be proven analytically for the CCR case, with one input and one output. Second, to obtain the input and output data, a provider and customer opinion survey is necessary. Finally, the presented methodology also has to allow deterministic inputs and/or output data to be considered.

This statistical efficiency methodology, used with opinion indexes from doctors and patients allows us to conclude that, in Spain, there are ten autonomous communities that can improve their efficiency and five autonomous communities that act as benchmarks for the rest.

The results of this paper can have other important implications in practice. It can also be used to measure the efficiency in all services with users and providers, for instance, markets, health care, banks, casinos, schools, universities or public transport.

## Proof of Lemma 1

The first equality is obtained by

35 $$ \begin{array}{@{}rcl@{}} && \left( \left|{\widehat{Y}}_{j}-Y_{j}\right|\le pY_{j} \right) =\left( \frac{{\widehat{Y}}_{j}}{(1+p)} \leq Y_{j} \leq \frac{{\widehat{Y}}_{j}}{(1-p)} \right) \end{array} $$

36 $$ \begin{array}{@{}rcl@{}} && \left( \left|{\widehat{X}}_{j}-X_{j}\right|\le pX_{j} \right) =\left( \frac{{\widehat{X}}_{j}}{(1+p)} \leq X_{j} \leq \frac{{\widehat{X}}_{j}}{(1-p)} \right) \end{array} $$

and, dividing the upper and lower extremes of Eq. 35 by the lower and upper extremes of Eq. 36, respectively, we obtain

37 $$  A_{j}=\left( \frac{(1-p)}{(1+p)}{\widehat{Z}}_{j}\le Z_{j}\le \frac{(1+p)}{(1-p)}{\widehat{Z}}_{j}\right). $$

The second equality is obtained by clearing ${\widehat {Z}}_{j},$ and the third by dividing by *Z*_*m*_.

### Proof of Lemma 2

If *m* = *l*, from Lemma 1, it follows that *A*_*l*_ = *B*.

If *m*≠*l*, then ${\widehat {Z}}_{m}<{\widehat {Z}}_{l}$ and *Z*_*l*_**<***Z*_*m*_ and, if *A*_*l*_ ∩ *A*_*m*_ is verified, it follows that:

$$\frac{\left( 1-p\right) }{(1+p)}Z_{m}\le {\widehat{Z}}_{m}<{\widehat{Z}}_{l}\le \frac{(1+p)}{(1-p)}Z_{l}<\frac{(1+p)}{(1-p)}Z_{m}.$$

### Proof of Lemma 3

Dividing the first inequality that defines the set *B* by $\left (1+p\right )$ and multiplying by $\left (1-p\right )$, we obtain:

$$\frac{(1-p)^{2}}{\left( 1+p\right)^{2}}\le \frac{{\widehat{Z}}_{l} (1-p)}{Z_{m}\left( 1+p\right)}$$

Now, for $j=1,{\dots } ,L$, as $ {\widehat {\varphi }}_{j}=\frac {{\widehat {Z}}_{j}}{{\widehat {Z}}_{l}} $, we have that:

$$ {\widehat{\varphi }}_{j}\frac{(1-p)^{2}}{\left( 1+p\right)^{2}}\le {\widehat{\varphi }}_{j}\frac{{\widehat{Z}}_{l}(1-p)}{Z_{m}\left( 1+p\right)}=\frac{{\widehat{Z}}_{j}(1-p)}{Z_{m}\left( 1+p\right)}; $$

Dividing the second inequality that defines the set *B* by (1 − *p*) and multiplying by (1 + *p*), we have:

$$\frac{{\widehat{Z}}_{l}(1+p)}{Z_{m}\left( 1-p\right)}\le \frac{(1+p)^{2}}{\left( 1-p\right)^{2}}.$$

Given that $j=1,{\dots } ,L$: we get ${\widehat {\varphi }}_{j}\frac {{\widehat {Z}}_{l}(1+p)}{Z_{m}\left (1-p\right )}\le {\widehat {\varphi }}_{j}\frac {\left (1+p\right )^{2}}{\left (1-p\right )^{2}}$ and, as $ {\widehat {\varphi }}_{j}=\frac {{\widehat {Z}}_{j}}{{\widehat {Z}}_{l}}$, we get $ \frac {{\widehat {Z}}_{j}(1+p)}{Z_{m}\left (1-p\right )}\le {\widehat {\varphi }}_{j}\frac {\left (1+p\right )^{2}}{\left (1-p\right )^{2}}$.

As from Lemma 1, $A_{j}=\left (\frac {{\widehat {Z}}_{j}(1-p)}{Z_{m}\left (1+p\right )}\le {\varphi }_{j}\le \frac {{\widehat {Z}}_{j}(1+p)}{z_{m}\left (1-p\right )}{\mathbf \ }\right )$, then, whenever *A*_*j*_ ∩ *B* happens, we have

$$ \left( {\widehat{\varphi }}_{j}\frac{(1-p)^{2}}{\left( 1+p\right)^{2}}\le \frac{{\widehat{Z}}_{j}(1-p)}{Z_{m}\left( 1+p\right)}\le {\varphi }_{j}\le \frac{{\widehat{Z}}_{j}(1+p)}{Z_{m}\left( 1-p\right)}\le {\widehat{\varphi }}_{j}\frac{(1+p)^{2}}{\left( 1-p\right)^{2}}\right). $$

### Proof of Theorem 1

$$ \begin{array}{@{}rcl@{}} P\left( {\widehat{\varphi }}_{j}\frac{\left( 1-p\right)^{2}}{\left( 1+p\right)^{2}}\le {\varphi }_{j}\le {\widehat{\varphi }}_{j}\frac{\left( 1+p\right)^{2}}{\left( 1-p\right)^{2}}\right)&\!\ge\!& P\left( A_{j}\cap B\right)\\ &\!\ge\!& P\left( A_{j}\cap A_{l} \cap A_{m}\right)={\left( 1-\alpha \right)}^{3} \end{array} $$

The first inequality follows from Lemma 3, the second from Lemma 2, and the last inequality follows because the events *A*_*j*_ are independent.

### Proof of Lemma 4

If ${\widehat {\varphi {}}}_{j}\frac {\left (1-p\right )^{2}}{\left (1+p\right )^{2}}\leq {}{\varphi {}}_{j}\Longrightarrow {}{\widehat {\varphi {}}}_{j}\left (1-\frac {4p}{(1+p)^{2}}\right )\leq {}{\varphi {}}_{j}\Rightarrow {}{\widehat {\varphi {}}}_{j}-{\varphi {}}_{j}\leq {}\frac {4p}{(1+p)^{2}}{\widehat {\varphi {}}}_{j}\leq {}\frac {4p}{(1+p)^{2}}$; the last inequality follows from $0\leq {}{\widehat {\varphi {}}}_{j}\leq {}1.$

If $min\left \{1,{\widehat {\varphi {}}}_{j}\frac {(1+p)^{2}}{\left (1-p\right )^{2}}\right \}=1\Longrightarrow {}{\widehat {\varphi {}}}_{j}\frac {\left (1+p\right )^{2}}{\left (1-p\right )^{2}}\geq {}1\Longrightarrow {}{\widehat {\varphi {}}}_{j}\geq {}\frac {\left (1-p\right )^{2}}{\left (1+p\right )^{2}}\Longrightarrow {}{\widehat {\varphi {}}}_{j}+\frac {4p}{(1+p)^{2}}\geq {}\frac {\left (1-p\right )^{2}}{\left (1+p\right )^{2}}+\frac {4p}{\left (1+p\right )^{2}}=1$ and $min\left \{1,{\widehat {\varphi {}}}_{j}+\frac {4p}{(1+p)^{2}}\ \right \}=1$.

If ${\varphi {}}_{j}\leq {}min\left \{1,{\widehat {\varphi {}}}_{j}\frac {(1+p)^{2}}{\left (1-p\right )^{2}}\right \}={\widehat {\varphi {}}}_{j}\frac {(1+p)^{2}}{\left (1-p\right )^{2}}$$\Longrightarrow {}{\varphi {}}_{j}\leq {}{\widehat {\varphi {}}}_{j}\left (\frac {1}{1-\frac {4p}{\left (1+p\right )^{2}}}\right )\Longrightarrow {}{\varphi {}}_{j}\left (1-\frac {4p}{\left (1+p\right )^{2}}\right )\leq {}{\widehat {\varphi {}}}_{j}\Longrightarrow {}{\varphi {}}_{j}-{\widehat {\varphi {}}}_{j}\leq {}\frac {4p}{\left (1+p\right )^{2}}{\varphi {}}_{j}\leq {}\frac {4p}{(1+p)^{2}}$; the last inequality follows from 0 ≤ *φ*_*j*_ ≤ 1.

## Proof of Theorem 2

The result follows from Theorem 1, Lemma 4 and $P(A_{j})=P\left (\left \vert {\widehat {X}}_{j}-X_{j}\right \vert {}\leq p X_{j}\right ) \cap \left (\left \vert {\widehat {Y}}_{j}-Y_{j}\right \vert {}\leq {p}Y_{j}\right ))=P\left (\left \vert {\widehat {X}}_{j}-X_{j}\right \vert {}\leq p X_{j}\right ) P\left (\left \vert {\widehat {Y}}_{j}-Y_{j}\right \vert {}\leq {p}Y_{j}\right ).$

## Appendix II

The software to reproduce the methods is available in the following link https://uvaes-my.sharepoint.com/:f:/g/personal/jesus_tapia_uva_es/EpnYVaKm1OZMpKUyyrI-bS0BNUj7aDumIquTR7Ipxt422A?e=GvrDtG

This file contains the following programs:

– Program to take survey sampling without replacement ``mas.m”
– Program to take survey sampling with replacement ``mascon.m”
– Programs to obtain the sample size: ``masnp.m” ``masn.m” ``masnvar.m”
– Program to obtain efficiency bootstrap confidence intervals ``percboot2outp2inp2pob.m”
– Program to obtain efficiency CCR ``ccrout.m”
– Program to obtain efficiency BCC ``bccout.m”
– Program to obtain the intervals and the results of the simulation ``CIE15ybootalfab01.m”
– Simulation data ``datcoop2i2ost2pob.mat”
